# Supplementary material for: The enhancement of CCL2 and CCL5 by human bone marrow-derived mesenchymal stem/stromal cells might contribute to inflammatory suppression and axonal extension after spinal cord injury
Source: PLoS One. 2020 Mar 10;15(3):e0230080. doi: 10.1371/journal.pone.0230080 (PMC7064230; doi:10.1371/journal.pone.0230080)

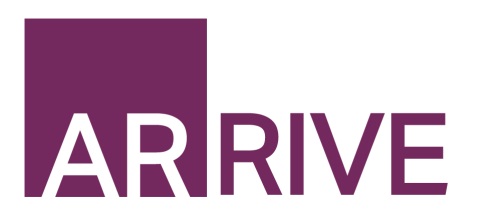


The ARRIVE Guidelines Checklist

Animal Research: Reporting In Vivo Experiments

Carol Kilkenny^1^, William J Browne^2^, Innes C Cuthill^3^, Michael Emerson^4^ and Douglas G Altman^5^

*^1^The National Centre for the Replacement, Refinement and Reduction of Animals in Research, London, UK, ^2^School of Veterinary Science, University of Bristol, Bristol, UK, ^3^School of Biological Sciences, University of Bristol, Bristol, UK, ^4^National Heart and Lung Institute, Imperial College London, UK, ^5^Centre for Statistics in Medicine, University of Oxford, Oxford, UK.*

|  | | ITEM | RECOMMENDATION | Section/ Paragraph |
| --- | --- | --- | --- | --- |
| 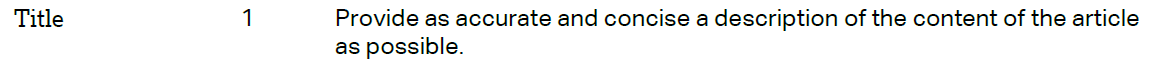 | | | Title |  |
| 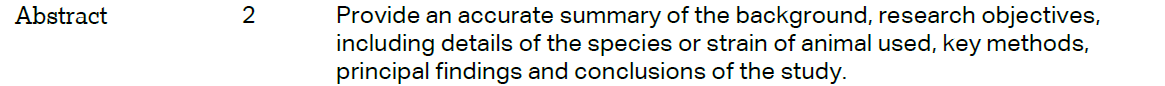 | | | Abstract |  |
| INTRODUCTION | | |  |  |
| 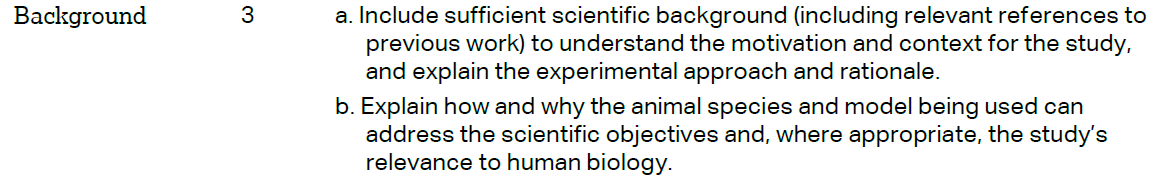 | | | Introduction |  |
| 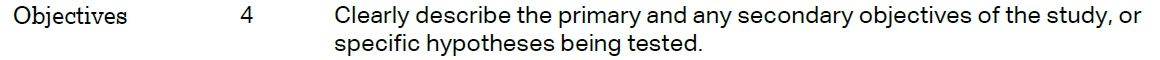 | | | To clarify how hMSCs communicate with or regulate MG/MΦ (Introduction, page5/line 9-21). |  |
| METHODS | | |  |  |
| 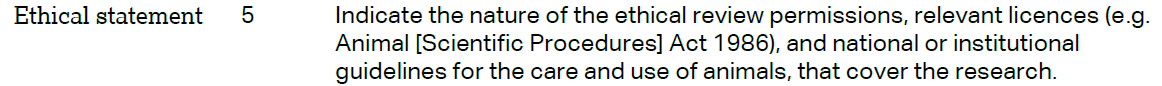 | | | Materials and methods, Animals section |  |
| 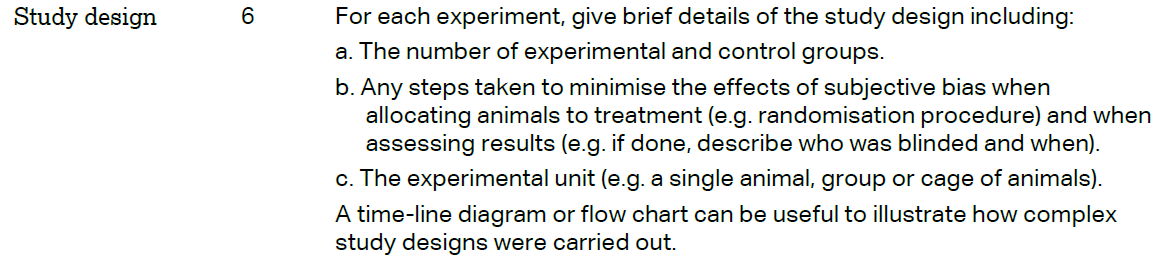 | | | a. Total of 139 animals were used for the study. The number of animals used in each experiment was listed in each figure legend.  b. Materials and methods, Statistical analysis section  c. SCI control group, hMSCs or HBSS injected group and RANTES or vehicle injected group. |  |
| 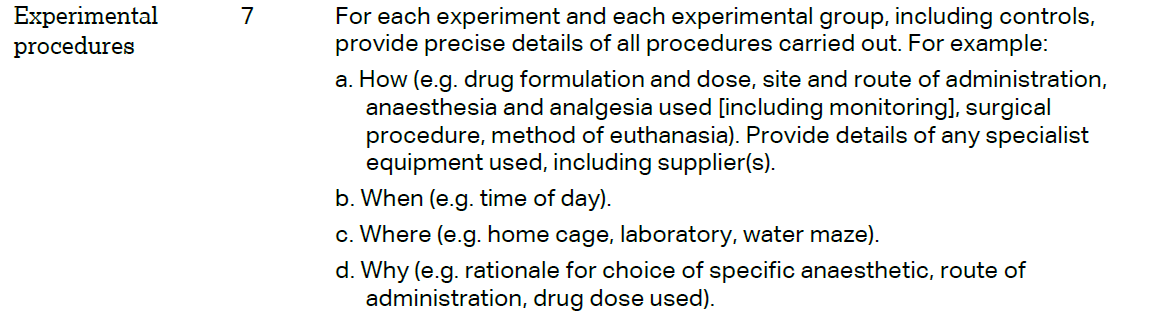 | | | Materials and methods, SCI model, Injection of hMSCs into the spinal cord, Isolation of RNA and production of cDNA, Immunostaning, and Injection of recombinant mouse CCL5 (RANTES) into the spinal cord sections. |  |
| 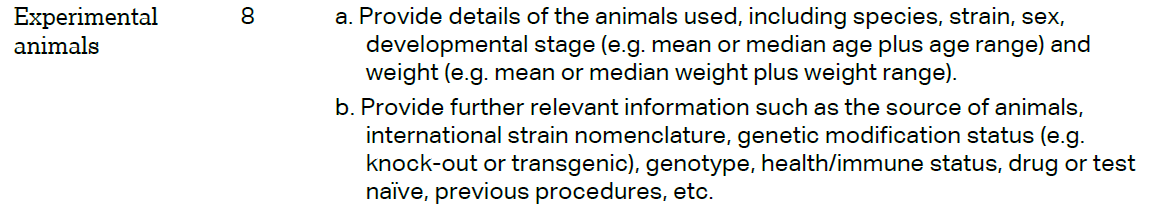 | | | Materials and methods, Animals section |  |

The ARRIVE guidelines. Originally published in *PLoS Biology*, June 2010^1^

| 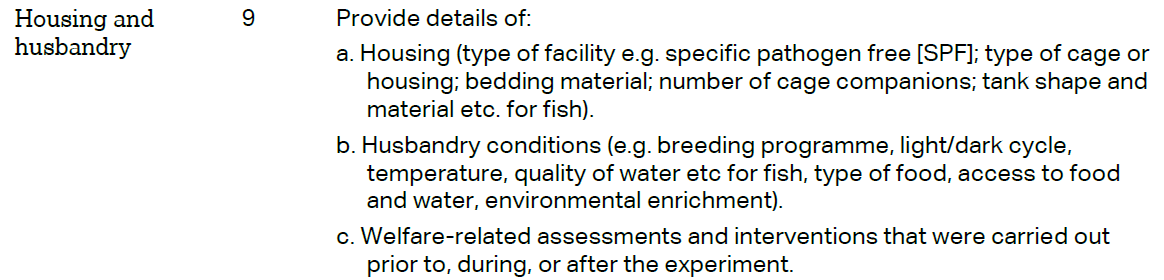 | a. 6 to 8 animals were kept in the same plastic cages. SPF grade.  b. Materials and methods, Animals section  c Materials and methods, SCI model section | |
| --- | --- | --- |
| 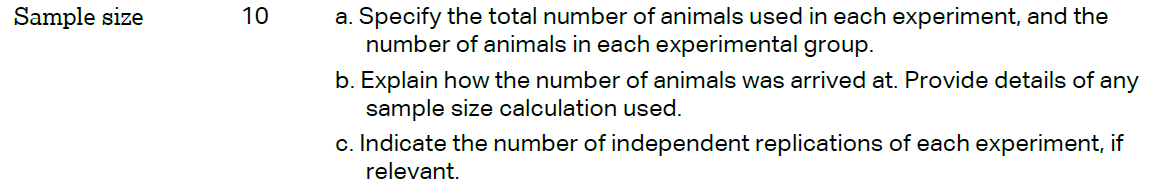 | a. Total of 139 animals were used for the study. The number of animals used in each experiment was listed in each figure legend.  b. Sample size was determined following previous study.  c. 6 to 8 animals were subjected to SCI at a time, and MSC or RANTES were injected into spinal cord on the way. Therefore, the experiment was divided into about 30 times in total. | |
| 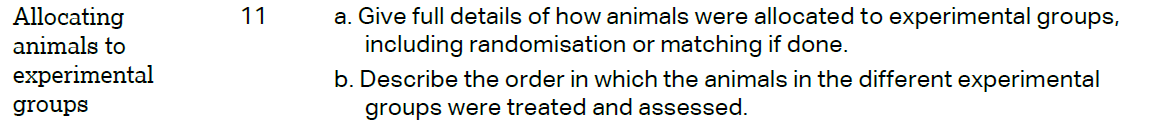 | Materials and methods and Results | |
| 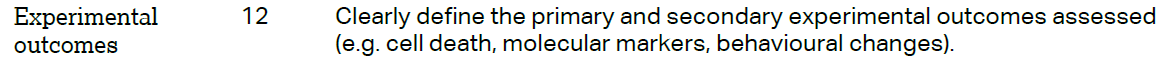 | Functional recovery, change of gene expression levels and localization of chemokines and receptors after hMSCs transplantation to SCI mice. | |
| 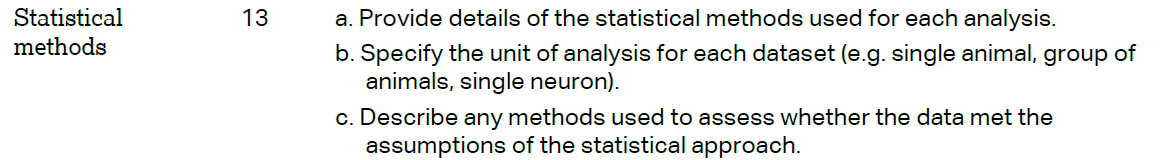 | a. Materials and methods, Statistical analysis section  b. All data that performed statistic analysis in the study were in groups.  c. Materials and methods, Statistical analysis section | |
| RESULTS |  | |
| 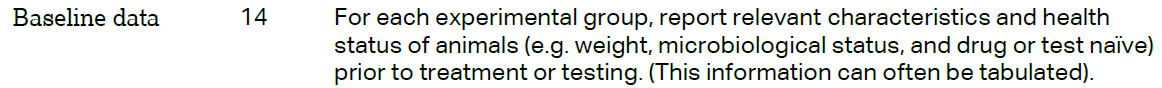 | Materials and methods, Animals section | |
| 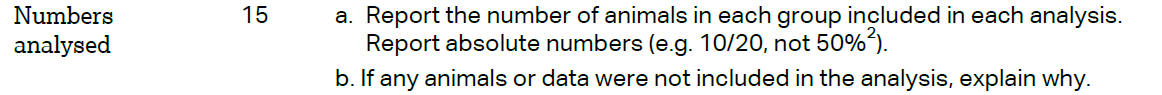 | a. SCI control group 45/132, hMSCs injected group 40/132, HBSS injected group 26/132, RANTES injected group 6/132, vehicle injected group 6/132, animals used for examination of retention of hMSCs 4/132 (Fig 4A), animals used for standard curve for semi-quantification of the number of hMSCs 5/132 (Materials and methods, Semi-quantification of the number of hMSCs in the spinal cord section).  b. Materials and methods, Assessment of motor function section | |
| 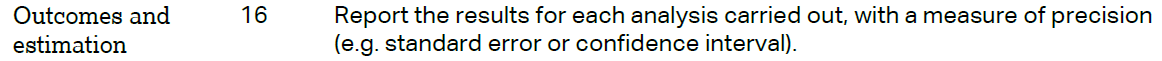 | Each figure legends | |
| 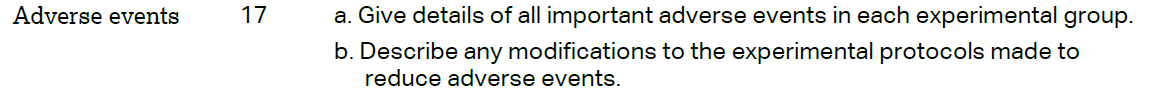 | a. SCI animals exhibited lower limb paralysis and dysuria.  b. To support urination, the region of the lower abdomen in all mice was gently stimulated a few times every day. | |
| DISCUSSION |  | |
| 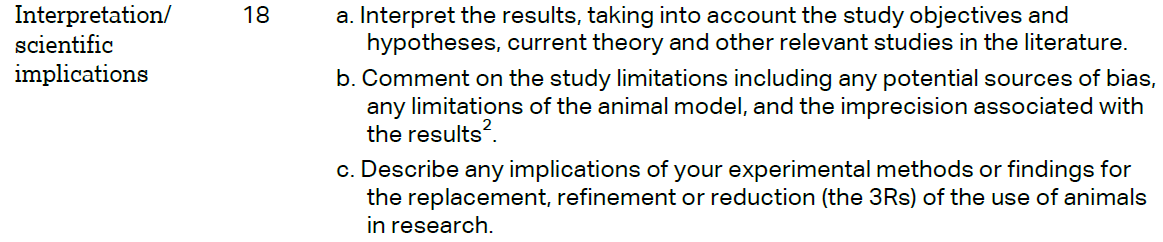 | Discussions | |
| 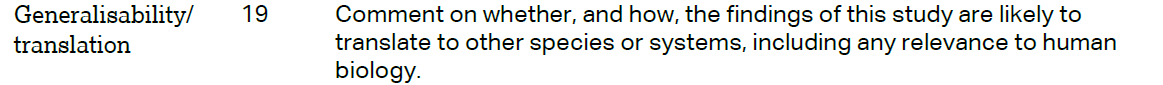 | Discussions | |
| 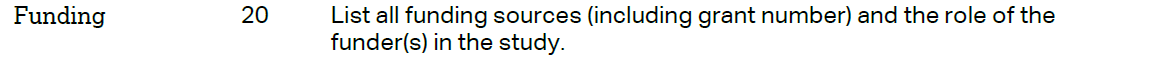 | | Decralations and Author contributions |


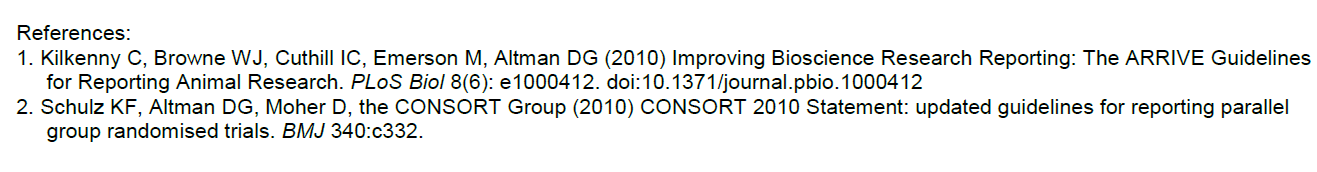

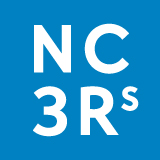

Supplement: S1 Checklist — (DOCX) [file pone.0230080.s001.docx]
