# Supplementary material for: The enhancement of CCL2 and CCL5 by human bone marrow-derived mesenchymal stem/stromal cells might contribute to inflammatory suppression and axonal extension after spinal cord injury
Source: PLoS One. 2020 Mar 10;15(3):e0230080. doi: 10.1371/journal.pone.0230080 (PMC7064230; doi:10.1371/journal.pone.0230080)
Supplement: Attachment — Submitted filename: Response to Reviewers (PONE-D-19-28233R1).docx [file pone.0230080.s002.docx]

Thank you very much for the academic editor’s and reviewer’s comments to improve our manuscript of paper (PONE-D-19-28233). We read the comments well, and answered and revised according to your suggestions. We also removed all the term “data not shown” as journal requirements. Although we have shown our ethics statement both in sub-section “Animals (see page 8, line 7~)” of the Materials and Methods, and in the other section “Ethics approval and consent participate (previously, page 62, line 2~), we removed the latter.

Comments from editor

*1. Provide evidence that hMSCs are present at the injection site*

*2. What is the survival rate and where are they located if they are not at the injury site*

Thank you very much for your comments. We thought that the comments 1 and 2 linked each other. So, we answered them together.

Injected hMSCs were disappeared quickly from the injective site. However, it is known to rescue several diseases (Prockop DJ. Clin Pharmacol Ther. 2007; <https://ascpt.onlinelibrary.wiley.com/doi/full/10.1038/sj.clpt.6100313>).

Also. in present study, we determined the fate of hMSCs using human GAPDH mRNA (*hGAPDH*) expression in the mouse spinal cord by real-time PCR (Fig. 1A). The estimate number of hMSCs was calculated by standard curve of the gene expression. Standard curves were generated by adding serial dilutions of hMSCs (1 × 10^2^ to 1 × 10^6^ cells) to spinal cord samples (T7 to T12 vertebrae) of uninjured mice prior to homogenization (see page 13, line 97~).

We have also reported previously the retention of hMSCs by *hAlu* genomic DNA expression and the localization of PKH26-labeled hMSCs by immunohistochemistry in same animal model (Fig. 2 in Tsumuraya et al. J Neuroinflammation 2015; <https://jneuroinflammation.biomedcentral.com/articles/10.1186/s12974-015-0252-5>). We showed in the study that the injected hMSCs are seen to have migrated along the spinal cord toward the injury site at 7 days after SCI.

The fate of hMSCs in the present study was shown in Fig. 1A. Immediately after hMSC injection (pod 1), approximately 260,000 hMSCs were detected (representing 50% of the injected cells) including the site of injection. The numbers of hMSCs decreased to approximately 40,000 cells on pod 3(15% of pod1), and have few-to-no hMSCs were detected on pod 14 (see page 21, line 10~). The reason, we choose *hGAPDH*, but not *hAlu* in present study is to eliminate a possibility of genomic DNA from hMSCs debris although the result was almost similar with previously.

We have reported the fate of hMSCs in our other studies of brain ischemia (Ohtaki et al. Proc Natl Acad Sci U S A. 2008; <https://www.pnas.org/content/105/38/14638.long>) and STZ-induced diabetes (Murai et al. PLoS One 2017; <https://journals.plos.org/plosone/article?id=10.1371/journal.pone.0186637>). These studies also support in the present results.

The question of “*where are they located if they are not at the injury site”* is very difficult to answer. In the present study, the cells as the *hGAPDH* level almost disappeared from the spinal cord on pod 14. The cells might eliminate by phagocytes and/or move to somewhere. We have examined the distribution of hMSCs in systemic organs by an *in vivo* fluorescence imager (Murai et al. PLoS One 2017). However, the detection of small amount of cells was extremely difficult. So, I am sorry we do not have accurate answer of this.

*3. Why are there no dendrites on neurons?*

In present study, we used anti-Neu N antibody as a neuronal marker. Neu N is known to be stained neuronal nuclei and the peri-nucleic region (nerve cell body), but not or few in fibers. So the staining of neurons seems to round and triangle sometimes.

Comments to reviewer

Thank you very much for your supportive comments. We revised our manuscript (indicated yellow highlight in the text) and commented to you below.

Major Comments:

*1. The main objective of the study needs to be clearly indicated and experiments need to coherently follow the main objective. How were the genes selected for the PCR experiment? Why choose CCL2/CCR2 and CCL5/CCR5?*

In present study was designed according to our previous studies. We have focused on the communication between transplanted hMSCs and microglia/macrophages (MG/MΦ) on SCI. Therefore, we firstly selected mainly the MG/MΦ including monocyte-related chemokines and the receptors. Then, from expressing pattern we further focused on the CCL2/CCR2 and CCL5/CCR5. CCL5/CCR5 is a chemokine and receptors increased at chronic periods. Although many chemokines (receptors) increased at acute periods, we selected the most popular MG/MΦ-related chemokine CCL2/CCR2 axis. We think that we should examine the other chemokines in the future study.

Although this is unpublished data, we are recognizing in another project that CCL2/CCR2 axis is an important signal to communicate hMSCs and recipient cells. Would you wait for wait a little more to reveal our next publication?

*2. The representation of expressional changes in Figure 1 needs to be revised. Line graphs or heatmaps would be more suitable for representing time-course transcriptional differences. How was the absolute PCR analysis performed?*

Thank you very much. We revised bar to line graphs in Fig.1 (now, this is Fig.2). Moreover, we have not described the unit in the text including the legend and graphs clearly. They are relative level (fold) to compare with day 0. This mentioned in the methods section (see page 14 line 125~) and figure legend. As described below, we reconsidered the order of the figures from your comment #3. Fig.1 in original manuscript changed to Fig.2.

*3. The functional recovery assessment is singular (relies only on BMS scores) and is terminated at 14 days while the difference between the treatment is continuing to deviate. The authors need to provide a justification for not continuing their analyses to the chronic phase.*

We have reported injection of hMSCs suppress neural injury through the macrophage or microglial modulation in the acute phase (within a week) after ischemia (Ohtaki et al. Proc Natl Acad Sci U S A. 2008). Moreover, the phenomenon has been reproduced after SCI as well (Tsumuraya et al. J Neuroinflammation 2015). Therefore, we decided the experiment periods based on the previous our studies and checked the reproducibility by BMS and gene expression of neuron specific enolase (Eno2) in the present study. However, we thought your comments also very reasonable if we showed figures in present order. We reconsidered again the order of the figures. We inserted Fig. 4 prior to Fig. 1 to be logically. Thank you for your supportive comments.

*4. The authors need to indicate how their transcriptional work in Figure 1 advances the previous studies performing transcriptional analyses in SCI research.*

Yes. Some of SCI studies have reported to the gene expression of chemokines. However, we have not determined the expressions in this model so far. Therefore, we did them.

*5. It’s not clear to the reader why the authors refrained from administration of immunosuppressants or immunodeficient animals, particularly considering the low survival of hMSCs following transplantation.*

Thank you for your comments. As your suggestion, many studies administer administration of immunosuppressants or use immunodeficient animals. However, to observe inflammation or immune systems, we were afraid that they might interfere to the systems. We have previously examined injection of hMSCs into brain parenchyma and compared the fate of hMSCs between immunodeficient and immunocompetent animals. Resulting, no significant differences were recognized (Ohtaki et al. Proc Natl Acad Sci U S A. 2008). Moreover, repetitive injections of hMSCs into immunocompetent animals did not show any anaphylactic reactions or die (Murai et al. PLoS One 2017). The following manuscript explain the reason to inject hMSCs into immunocompetent animals. Please see following paper (ref #10).

Prockop DJ, Oh JY, Lee RH. Data against a Common Assumption: Xenogeneic Mouse Models Can Be Used to Assay Suppression of Immunity by Human MSCs. Mol Ther. 2017 2;25(8):1748-1756. doi: 10.1016/j.ymthe.2017.06.004.

Minor Comments:

*6. The authors need to justify their choice for using a transection model for studying immune cell migration.*

To compare with contusion model, the transection model is localized the injury to a small region. Therefore, we thought to be clear cell-cell communications. Moreover, as the other reason, we continuously studied SCI using this model and there are some evidences for the model.

*7. The abstract needs to be reorganized and compressed. While the authors are informative and descriptive, they should succinctly describe their major findings and main inferences.*

Thank you. We revised.

*8. Line 25: “Central” should not be capitalized.*

Thank you. We revised.
