# Supplementary material for: The enhancement of CCL2 and CCL5 by human bone marrow-derived mesenchymal stem/stromal cells might contribute to inflammatory suppression and axonal extension after spinal cord injury
Source: PLoS One. 2020 Mar 10;15(3):e0230080. doi: 10.1371/journal.pone.0230080 (PMC7064230; doi:10.1371/journal.pone.0230080)
Supplement: Attachment — Submitted filename: Response to Reviewers (PONE-D-19-28233R2).docx [file pone.0230080.s003.docx]

Thank you very much for the academic editor’s and reviewer’s comments to improve our manuscript of paper (PONE-D-19-28233). We read the comments well, and revised according to your suggestions.

Comments to reviewer

*1. The manuscript is much improved. However, grammar mistakes and the writing style at the end of the introduction should be addressed. Additionally, the authors may want to mention the justification for the transection model and not administering immunosuppressants in the discussion.*

Thank you very much for your supportive comments. We revised our manuscript (indicated yellow highlight in the text) according to your comments.

Moreover, we checked entire of manuscript again and slightly changed the order of references.
